# Analysis of Enantiomers in Products of Food Interest

**DOI:** 10.3390/molecules24061119

**Published:** 2019-03-21

**Authors:** Chiara Fanali, Giovanni D’Orazio, Alessandra Gentili, Salvatore Fanali

**Affiliations:** 1 Department of Medicine, University Campus Bio-Medico of Rome, Via Alvaro del Portillo 21, 00128 Rome, Italy; 2 Istituto per I Sistemi Biologici, Consiglio Nazionale delle Ricerche, Via Salaria km 29, 300-00015 Monterotondo, Italy; giovanni.dorazio@cnr.it; 3 Department of Chemistry, University of Rome “La Sapienza“, Piazzale Aldo Moro 5, P.O. Box 34, Posta 62, 00185 Roma, Italy; alessandra.gentili@uniroma1.it; 4 Teaching Committee of Ph.D. School in Natural Science and Engineering, University of Verona, 37134 Verona, Italy; salvatore.fanali@gmail.com

**Keywords:** chiral, chiral stationary phases, enantiomers, food, review

## Abstract

The separation of enantiomers has been started in the past and continues to be a topic of great interest in various fields of research, mainly because these compounds could be involved in biological processes such as, for example, those related to human health. Great attention has been devoted to studies for the analysis of enantiomers present in food products in order to assess authenticity and safety. The separation of these compounds can be carried out utilizing analytical techniques such as gas chromatography, high-performance liquid chromatography, supercritical fluid chromatography, and other methods. The separation is performed mainly employing chromatographic columns containing particles modified with chiral selectors (CS). Among the CS used, modified polysaccharides, glycopeptide antibiotics, and cyclodextrins are currently applied.

## 1. Introduction

In recent years, in the field of separation science, there has been great attention to analyzing products of food interest. All this has been done to meet the needs of both the industries operating in the field and the control laboratories belonging to the various national and international agencies. The use of modern and reliable analytical methods permits knowing the chemical composition of these products allowing, e.g., to determine their quality, to trace problems related to production, and storage processes, etc. In addition, the presence of dangerous pollutants such as pesticides present in foodstuff and/or additives can also be quantified.

A large number of compounds present in food products have been successfully examined, e.g., amino acids, proteins, carbohydrates, vitamins, lipids, mycotoxins, colorants, preservatives, herbicides, ionic compounds, fungicides, and enantiomers, etc.

Among all the classes of compounds analyzed so far, particular attention has been paid to the separation and determination of chiral compounds. Two enantiomers have the same chemical composition with, e.g., one asymmetric center, and due to the different spatial orientation of the substituent groups, they exhibit quite similar physical-chemical properties. However, due to the chirality, they can participate, with a different effect, to the various biochemical processes. In these processes, enantiomers could react differently with, e.g., enzymes, proteins, and peptides, etc., present in humans determining beneficial or dangerous effects. In addition, natural products must contain only one enantiomer as in the case of fruit juices where only the *L*-amino acid must be present. The existence of the antipode can indicate either an adulteration (addition of a racemate) or other problems related to poor storage. Therefore, it is very important to have reliable analytical methods able to determine qualitatively and quantitatively the enantiomeric forms present in samples of food interest.

Analytical techniques so far used for the analysis of enantiomers include: Gas chromatography (GC) [1], supercritical fluid chromatography (SFC) [2,3], thin layer chromatography (TLC) [4], high-performance liquid chromatography (HPLC) [5], capillary electrophoresis (CE), and capillary-/nano-liquid chromatography (CLC/nano-LC) [6,7], etc.

In this review, general principles of enantiomers separation utilizing chromatographic techniques and the most applied chiral stationary phases (CSPs) are reported and discussed. In addition, the most important applications of some analytical techniques to the analysis of chiral compounds in food matrices published in the last two years are presented.

## 2. General Separation Methods in Chiral Resolution

As previously mentioned, the very similar physical-chemical properties of two enantiomers are the main reasons for the difficulty in obtaining their resolution by using conventional methods such as, for example, reversed phase one in liquid chromatography. However, their separation can be easily achieved utilizing a chiral background.

Generally, two resolution methods can be used, namely indirect and direct ones. In the first procedure, the two compounds react with a chiral selector (CS) on forming stable diastereoisomers where strong bonds are formed. The two new compounds can be separated employing a non-chiral stationary phase (SP). The separation method offers some benefits, e.g., the introduction of additional groups useful for i) further interactions and ii) increase the sensitivity when UV and mass spectrometry (MS) detectors are applied. However, a high reagent purity is necessary and it is time-consuming [8].

The direct resolution method is the most applied in separation science. It is based on the use of a CSP where the two analytes interact continuously on forming diastereoisomeric complexes, and involving weak bonds (hydrogen, π-π, hydrophobic etc.). While some models have been proposed for chiral recognition, the “Three point” interaction could be considered in order to achieve compounds separation. However, enantiomers resolution can also be obtained when repulsion, in addition to interactions, are involved in the stereoselective mechanism [9].

**Figure 1:**
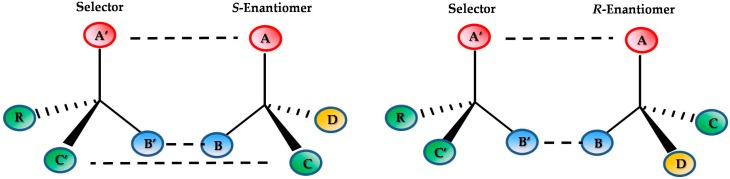
Scheme of “Three-point” interaction model.

Figure 1 shows a scheme of the “Three-point” interaction model.

Even if the number of CSs, applied to the resolution of enantiomers is quite large and varied, it is noteworthy to mention that it is not possible to find a universal one. They have been utilized employing different analytical techniques, e.g., GC, SFC, HPLC, CLC/nano-LC, and CE etc. [8,10,11,12,13].

Among all the chiral selectors used so far with the different analytical techniques, those that deserve special attention are peptides, chiral amino acids, cyclodextrins, polysaccharide derivatives, quinine-based, glycopeptides antibiotics, and chiral crown-ethers, etc.

## 3. The Most Used Chiral Stationary Phases for Enantiomers Separation

In this section, the most applied CSPs to the separation of enantiomers utilizing the analytical techniques above mentioned will be summarized. Some of them have been applied to the analysis of compounds of food interest. Over the years, a large number of CSPs has been prepared and studied for enantiomers separation. Several of them are commercially available under different trade name also containing the same CS. This is the case of Lux or Chiralpak by Phenomenex (USA) and Chiral Technologies (USA), respectively. They make use of modified polysaccharides either coated or immobilized on silica particles. In order to improve the enantioresolution capacity of this CS type, cellulose or amylose have been modified with phenylcarbamate groups containing substituents such as methyl, chloro, and bromo, etc., alone or in combination. The presence of these substituents with electron-withdrawing or electron-donating modifies the properties of the phenyl promoting different interactions with the enantiomers to be separated [14]. Concerning the interactions involved in the chiral recognition when polysaccharides are employed, hydrogen, π-π bonding have to be mentioned. The inert support is usually silica of different dimensions (sub-2 μm to 5μm) porous or recently core-shell [15,16]. Monolithic material has also been used [17,18]. These CSPs have been largely employed in HPLC [5,19], SFC [11,20], capillary electrochromatography (CEC) [21,22], and nano-LC [23].

Another CS, widely applied to enantiomers separation, deserving attention, includes cyclodextrins (CDs) or their derivatives. It can be either bonded to silica particles or monolithic material or rarely added to the mobile phase. In addition it can be coated on the capillary wall, as used in GC [24]. CDs or their derivatives have been widely used in capillary electrophoresis added to the background electrolyte [12]. CDs are oligosaccharides with a shape like a truncated cone with a hydrophobic cavity and a hydrophilic outside (presence of hydroxyl groups). The recognition mechanism is based on inclusion complexation where the two enantiomers fit into the cavity. Steresoselective interactions with hydroxyl or substituent groups (e.g., methyl, ethyl, and hydroxypropyl, etc.) take place. However, adsorption interactions could also be involved, especially if organic solvents are employed in the mobile phase. Schurig has showed a unified enantioselective approach for enantiomers separation. Here the same capillary column (50 μm i.d.) containing a permethylated-β-CD, thermally coated, has been employed in both open-GC, -SFC, -LC, and –CEC [25].

Among other CS used for enantiomers separation, chiral crown-ethers have a certain interest. These CSP type are commercially available, e.g., crownpakcr(+) and cr(−) (Chiral technologies, USA) or Larihc CF6- P (AZYP, USA). The second column contains cyclofructans groups forming a basket structure based on crown-ether. Therefore, enantiomers enter into the basket and on the contrary of CDs, the hydrophilic groups interact with the cavity. This CSP type has been used for the enantiomers separation of compounds containing amino groups in their structure (amino acids, amines, and derivatives). Recently Armstrong’s group reported the screening of 119 primary amine-containing compounds achieving enantioresolution for 92% of analytes [26]. Finally, it is noteworthy to mention the use of ion-exchange type CSP [27,28,29,30] and those containing glycopeptides antibiotics (vancomycin, teicoplanin, etc.). All of them are commercially available columns offering good/high enantioselectivity towards a large number and type of compounds [31,32,33,34,35].

Glycopeptide antibiotics contain in their chemical structure a consistent number of asymmetric centers, amino, amide, aromatic, carboxylic groups offering different interaction types with the analyzed enantiomers. The recognition mechanism is based on the affinity interaction strongly influenced by the type of mobile phase, pH, ionic strength, organic solvent type, and concentration, etc. They have been used bonded to either porous or core-shell particles (1.7–5 μm diameter) [23,36].

## 4. Some Selected Applications to Enantiomers Separation in Food Chemistry

This section describes the recent data present in the literature reported in 2017–2018 and related to the enantiomers separation in food samples. Data on this topic, related to previous years, can be found in previous reviews [23,37].

### 4.1. Supercritical Fluid Chromatography

Although SFC is not a recent analytical technique, it has enormous potential for both analytical and preparative purposes. Among its main features, we can mention: Fast balance of the columns, high efficiency, and use of non-hazardous solvents. Making use of stationary phases, developed for HPLC applications, SFC has also been applied to the separation of enantiomers. Among these CSPs, those based on polysaccharides, cyclodextrins, vancomycin, and Pirkle-type are the most employed ones. The mobile phase contains carbon dioxide often modified with some organic solvents such as methanol, ethanol, isopropanol, and acetonitrile at relatively low concentrations. In addition to enhancing chiral resolution, other additives, e.g., formic acid (FA), acetic acid (HAc), trifluoracetic acid (TFA), ammonia (NH_3_), and diethylamine (DEA) have been used [37].

As can be observed in Table 1 six papers, dealing with enantiomers separation by SFC in samples of food interest, appeared in the considered last two years. In the developed analytical methods, chiral resolution was obtained utilizing CSP based on vancomycin and amylose or cellulose derivative.

**Figure 2:**
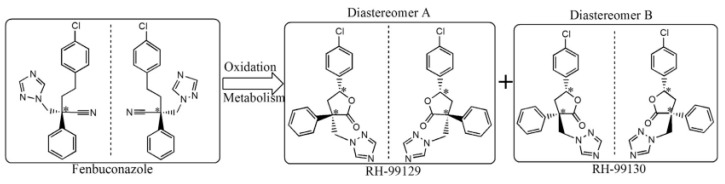
Chemical structure of fenbuconazol enantiomers and its metabolite diastereoisomers. Reproduced with permission of Elsevier from ref. [39].

Prothioconazole is a triazole fungicide widely used for its curative and protective action. Its enantiomers have been separated and analyzed by Jiang et al. [38] by using SFC. Analytes were extracted in a food sample (tomatoes) applying the Quick Easy Cheap Effective Rugged Safe (QuEChERS) sample preparation method and analyzed in a silica column coated with a modified cellulose derivative. Chiral separation was achieved in less than four minutes with good precision, accuracy, and recovery. In this study, method optimization was done by investigating some experimental parameters, e.g., the different type of polysaccharide columns, the composition of the mobile phase (carbon dioxide with additives such as methanol, ethanol, isopropanol-IPA). Best results were obtained using IPA as an additive at 30% (*v*/*v*) concentration. Another CS, namely amylose tris-(3,5-dimethylphenylcarbamate), coated on silica particles was used for the chiral separation of fenbuconazol and its metabolites by SFC. Analytes were also characterized by using MS/MS. Fenbuconazole is a compound with fungicidal activity, currently used for the treatment of fruits and vegetables. Two of its degradation lactone metabolites could contaminate groundwater and therefore their determination is of great interest. Very good enantiomers separation was obtained by SFC using a silica column coated with amylose tris-(3,5-dimethylphenylcarbamate) trough CO_2_/ethanol applying a gradient elution mode. Sample preparation was done employing a QuEChERS method. The six stereoisomers were separated in less than four min. In order to increase the MS ionization, 0.1% (*v*/*v*) formic acid/methanol compensation solution was added. The optimized method was validated with various samples, e.g., tomatoes, cucumbers, apples, peaches, rice, and wheat. Application to a real sample (greenhouse cucumbers) revealed the presence of only the two enantiomers of fenbuconazol [39]. Figure 2 reports the chemical structure of the two enantiomers of fenbuconazol and its diastereoisomers.

Another polysaccharide based column (tris(3,5-dimethylphenylcarbamoyl) cellulose) was used for analysis of triticonazole enantiomers in cucumbers and tomatoes with the same method. The separation was achieved in three min (in HPLC, eighteen min) eluting with CO_2_-ethanol (80:20, *v*/*v*) [40]. Four stereoisomers of propiconazole, a widely used triazole fungicide, were separated by SFC using different polysaccharide SPs. Among them, Chiralpak AD3 and Chiralpak IA3 allowed the separation of all stereoisomers in less than five min [41]. The two SPs contained amylose modified with tris(3,5-dimethylphenylcarbamate) coated and immobilized, respectively. Retention order was assessed by measuring the optical rotation of the four studied compounds. The optimized method was validated and applied to extract samples of wheat, grape, and soil matrices with good results concerning recovery.

### 4.2. High-Performance Liquid Chromatography

Selected applications to the analysis of chiral compounds utilizing HPLC are reported in Table 2 and described in this section.

Clenbuterol is a β_2_ agonist that is used in the treatment of respiratory disorders in humans. However, this drug, in some countries, even if forbidden, is also administered to the animals. The two enantiomers have been recently separated utilizing both HPLC and SFC employing columns containing vancomicin or teicoplanin [42]. Baseline separation of the two enantiomers was obtained with both techniques, however, a shorter analysis time was observed utilizing SFC (3.5 and 6 min, respectively). The methods were applied to the analysis of meat samples and urines of humans after eating the meat. Cattle meat contained enriched *R*-(−)-clenbuterol. More recently, the two analytical techniques have also been used by Chen and Zhang [43] for the chiral resolution of some isobutylhydroxyamides. Different commercial columns, amylose-based have been employed. While analyzed compounds belonging to the same class, two different techniques and different columns had to be used. These compounds are quite important in the field of phytochemistry and traditional Chinese medicine; they are present in Sichuan pepper. In this study, authors separated and characterized several isobutylhydroxyamides enantiomers also studying protective effects (PC12 Cells), reporting that compounds with *R* configuration resulted protective on the contrary of the *S* form.

Vegetables such as spinach, tomatoes, and cucumber have been analyzed for the determination of *R* and *S*-titriconazol using HPLC. This compound belongs to the family of fungicides used in agriculture. In this study, the single enantiomers and the racemic mixture were used and the bioactivity was investigated. The analyzed vegetables contained a high concentration of titriconazol after foliar spraying. However, the concentration of the two enantiomers decreased along the time. The *R*-isomer was dissipated more than its antipode. Therefore, it can be concluded that using the single enantiomer (*R*-) would reduce potential health risk [44]. A different fungicide, pyrisoxazole was studied by Yang et al. [45]. Enantiomers were separated with a commercial cellulose tris(4-methylbenzoate) column by HPLC in less than 10 min. This fungicide contains two asymmetric centers and therefore two couples of enantiomers. A similar study was carried out by Wang et al. [46] to study the degradation of isofenphos-methyl, a pesticide, after treating cowpea, cucumber, and pepper.

The two enantiomers were analyzed by HPLC and detected with MS/MS utilizing a commercial cellulose-based column (modified with 3-chloro-4-methylphenylcarbamate). Based on the presented results, the type of vegetable strongly influenced the degradation process, e.g., (*R*)-(−)-isofenphos-methyl was faster than (*S*)-(+)-isofenphos-methyl in cowpea and cucumber, while the opposite was observed in pepper. While showing interesting results, the authors did not further report about the reasons for the differences found.

Recently, an interesting HPLC method was studied utilizing cellulose or amylose 3, 5-dimethylphenylcarbamate for the separation of the four isomers of metconazole. An optimum resolution was achieved, after studying various mobile phases, employing the cellulose-based column and eluting with an organic solvent (hexane) with 3%, *v*/*v* of ethanol. The method was validated and applied to the analysis of the four compounds in flour. While the proposed method was carefully optimized studying the effect of various parameters on the compounds separation, the real sample was spiked and there was no finding of the transformation of the enantiomers/diastereoisomers [47]. 

The use of an HPLC method, employing polysaccharide-based columns (cellulose or amylose derivatives) allowed the chiral resolution of Thiols 3-sulfanylhexan-1-ol (**1**) and its O-acetate, 3-sulfanylhexyl acetate (**2**) after derivatization with 4-thiopyridine. The two compounds are quite important in assessing wine quality because of their potent aroma properties. In order to increase the MS signal and to have additional interaction groups for stereoselective interactions with the SP, the derivatization was helpful. Concerning enantiomers, the authors found that dry wines contained the two enantiomers of **1** and **2** at the same concentration, while in botrysed wines, elevated concentrations of *S*-form were found [48].

Chiralpak AD-H was used for semipreparative purposes for the enantiomers separation of some neolignans present in raspberry. A quantity of 2.5 mg of each enantiomer was obtained and the compounds investigated as potential inhibitors of β-amyloid aggregation. The study could be interesting in the field of nutraceutical research [49]. As reported above, all studies have been carried out using CSP polysaccharides bases, however, other CSs have also been used.

The enantioselective dissipation of an herbicide (fluazofop-butyl) was also studied in some vegetables (tomato, cucumber, pakchoi, and rape). The compound was degraded to fluazifop. Enantiomers separation was carried out in a commercial column packed with silica with immobilized cellulose tris(3,5-dichlorophenylcarbamate). As a result, the authors reported that the type of vegetable influenced the different behavior of the two chiral herbicides *S*-fluazifop-butyl dissipated faster than its antipode, while the contrary was observed in pakchoi, rape. The different behavior accounted for the presence of enzymes and to some chiral endogenous substances present in the plants [52]. 

α-, β-, and γ-hexabromocyclododecanes (HBCDs) are compounds used for different purposes, e.g., polystyrene foams, thermal insulation buildings, electric insulators, etc. Therefore they can be found in the environment and consequently in animals such as fishes, birds, chickens, etc. Enantiomers and diastereisomers, related to these compounds, have been separated by HPLC and the method applied to study the bioaccumulation in chicken tissues and eggs. Chiral compounds were separated using a permethylated-β-cyclodextrin silica column after recovering the different fractions subjected to sample preparation (soxhlet and gel permeation chromatography). It was observed that in adult chicken tissues (−)-α-HBCD and (+)-γ-HBCD were present at a higher concentration than the corresponding antipode [51].

A different chiral column containing α_1_-acid glycoprotein (AGP) has been employed for the enantiomers and diastereoisomers separation of chloramphenicol (CAP) in honey by HPLC. The analysis of this antibiotic is very important because it has been found in some foodstuff causing health problems. Due to the presence of two asymmetric centers, four stereoisomers are exhibited (*RR*- and *SS*-CAP; *RS*- and *SR*-CAP) [50].

Usually, amino acid enantiomers present in the natural product are abundant in their L-form, however, in some fermented food, various D-amino acids could be present. Therefore, the enantiomers analysis is very important in order to assess food quality and properties. Amino acid chiral separation has been carried out utilizing a chiral crown-ether column in chimchi (Chinese cabbage) [55], in black vinegar and yogurt [54], in vinegar [56]. The chiral separation of a large number of amino acids has been obtained without derivatization. Finally, it has been found that the concentration of D-amino acids increased during the storage.

A few papers reported the enantiomers separation utilizing ultra high-performance liquid chromatography (UHPLC). This is a powerful methodology where compounds are separated in columns packed with particles of small diameter (<2–3 μm). High selectivity, high chromatographic efficiency, and short analysis time are the main advantages of this technique. However, it is noteworthy mentioning that increased back-pressure is obtained (high-pressure pumps are necessary) [59,60,61]. This methodology has been applied to the separation and analysis of a fungicide compound (prothioconazole) and its metabolite prothioconazole-desthio. The analytes have been base-line resolved in their enantiomers using a cellulose- tris(4-methylbenzoate) column by HPLC, while no baseline resolution was observed with UHPLC [59].

**Figure 3:**
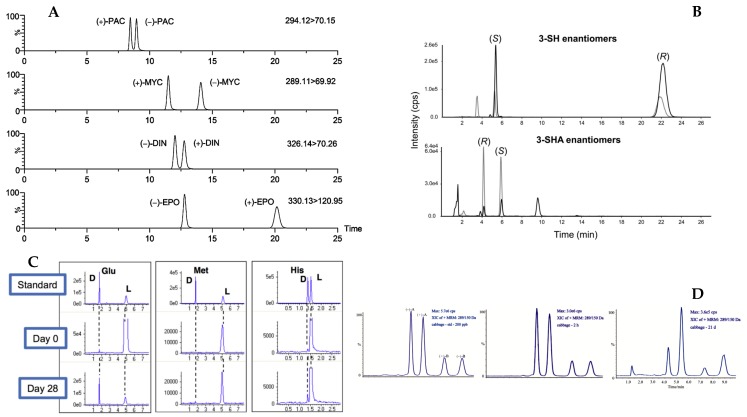
Typical enantioselective LC–MS/MS chromatograms: (**A**) chiral triazole fungicides in blank honey spiked using a Chiralcel OD-RH column modified with permission from [57]; (**B**) 3-sulfanylhexan-1-ol (3-SH 1) and 3-sulfanylhexyl acetate (3-SHA 2) isolated from a Sauvignon blanc wine on a CSP -Amylose-1 column; modified with permission from [48]. (**C**) Enantiomers resolution of some amino acids in kimchi stored for 25/28 days analyzed on a CSP- CROWNPAK CR-I by LC-TOF; modified with permission from [55]; and (**D**) Enatiomeric separation of fungicide pyrisoxazole by LC-MS on a CSP- Lux Cellulose-3; modified with permission from Reference [45].

Among other recently developed analytical technique, one of them, namely nano-liquid chromatography (nano-LC open tubular) has been proposed for the enantiomers separation of some amino acids in apple juice. Amino acids have been derivatized with fluorescein isothiocyanate (FITC) and separated in a fused silica capillary containing, on the wall, a thin layer of polymeric material (monolithic) modified with a chiral selector (β-cyclodextrin). The optimized method was applied to the analysis of amino acid enantiomers in apple juice. While the technique can offer some advantages over conventional ones, there are some limitations especially considering the sample loading and the limited amount of chiral selector present in the capillary [62]. Table 2 summarizes the data related to the use of HPLC for enantiomers separation in food products. Some representative chiral separations of compounds in food matrices by HPLC-MS are reported in Figure 3.

### 4.3. Gas Chromatography

Table 3 reports the application to the analysis of enantiomers in food products achieved by gas chromatography.

Finally, gas chromatography (GC), widely used for enantiomers resolution, can offer high chromatographic efficiency. It is applied mainly to volatile compounds. The separation is performed in a capillary of thin i.d. where a chiral selector is coated or bonded on the wall. In the publications appeared during this time, polymeric material, β-cyclodextrin-based has been used as a chiral selector. Pollutants such as 1,2,5,6,9,10-hexabromocyclododecane (HBCD) (six diastereisomeric couple of enantiomers) were studied by GC-MS in order to verify the bioaccumulation in fishes [63]. In order to verify whether the processing would influence the enantiomeric ratio, chiral terpenoids present in juice industry by-products have been analyzed with GC-MS by Marsol-Vall et al. [24]. Among the results, authors observed that monoterpene alcohols (terpinen-4- ol and α-terpineol) exhibited a change on the enantiomeric ratio concluding that this was probably due to the heat applied during the drying process. The same technique has also been applied to the analysis of enantiomers present in essential oil from thyme. In this study, authors found the presence of (−)-linalool, (−)-borneol and (+)-limonene concluding that these compounds could be useful to assess origin and authenticity of the product [64]. A more advanced analytical technique, namely two dimensional-GC-MS(2D-GC-MS) has been applied to the analysis of chiral compounds present in tea samples [66]. The study aimed to investigate the chiral stability of lemon-flavored hard tea during storage. Samples were firstly analyzed by GC in a DB-Wax column and then target compounds heart-cutted and online submitted to a chiral column (cyclodextrin-based) for chiral analysis. Authors found that *R*/*S* ratio of limonene did not change during storage, while at high-temperature, *S*-limonene increased. Linalool enantiomers analysis, at the beginning of storage, revealed the presence of a higher amount of *R*-enantiomer. During storage, the *R*/*S* ratio decreased due to the conversion of *S*-isomer to *R*- one. Similar results have been obtained for α-terpineol.

## 5. Conclusions

The separation of chiral compounds has received great attention also in the last few years in different application fields including food chemistry. HPLC, SFC, and GC are the most utilized techniques because their features employing essentially the direct resolution method. Chiral columns, containing particles modified with selected CSs allowed the enantiomeric resolution of a large number of compounds. The majority of the enantioseparations have been obtained using polysaccharide-type CSs because of their very high chiral selectivity. The presented applications in the field of food analysis document the need of powerful analytical methods to assess the role of enantiomers for safety and product origin. Further studies are needed in order to (i) develop new CSPs (ii) study modern analytical techniques such as UHPLC and nano-LC offering high efficiency and reducing analysis time.

## Figures and Tables

**Table 1:** Selected applications of supercritical fluid chromatography (SFC) to Food analysis.

Samples	Matrix	Sample Preparation	Technique	Column and Chiral Stationary Phase	Mobile Phase	Detection	References
(*R*) and (S)-Prothioconazole	Tomatoes	QuEChERS	SFC	cellulose tris(3,5-dimethylphenylcarbamate)-silica coated, EnantioPak (China) (150 × 4.6 mm, 5 µm)	CO_2_/2-propanol (80:20, *v*/*v*); 2.5. mL/min	UV, 254 nm	[38]
Fenbuconazole and metabolites	tomatoes, cucumbers, apples, peaches, rice and wheat,	QuEChERS	SFC	amylose tris-(3,5-dimethylphenylcarbamate)-coated chiral column	CO_2_/ethanol; 1.8 mL/min	MS/MS	[39]
Triticonazole	cucumbers and tomatoes	QuEChERS	SFC	tris(3,5-dimethylphenylcarbamoyl) cellulose-coated silica gel EnantioPak OD column (China) (150 mm × 4.6 mm, 5 µm)	CO_2_/ethanol (80:20, *v*/*v*)	-	[40]
Propiconazole	Wheat	Solid phase	SFC	Amylose tris(3,5-dimethylphenylcarbamate) and immobilized Amylose tris(3,5-dimethylphenylcarbamate), Chiralpak AD-3 and IA3, respectively (Daicel Chemical Industries, Japan) (150 mm × 4.6 mm i.d., 3 μm particle size)	CO_2_/ethanol (93:7, *v*/*v*)	MS/MS	[41]
Clenbuterol	Meat	Liquid-liquid extractionand Solid phase	SFC or HPLC	Vancomycin, Astecchirobiotic V2 (150 × 4.6 mm or 2.1 mm, particle size 5 µm); Teicoplanin, Astecchirobiotic T (150 × 4.6 mm or 2.1 mm, particlesize 5 µm);	CO_2_/ammonia or formic acid in SFC and MeOH (with water or ACN or 2-propanol) in HPLC	MS/MS	[42]
Isobutylhydroxyamides ZP-amide A and ZP-amide B	Pepper	Liquid-liquid extraction	SFC or HPLC	Amylose tris-(3,5-dimethylphenylcarbamate) coated, Chiralpak AD-H column (chiral column 1); Amylose tris(5-chloro-2-methylphenylcarbamate) coated, Chiralpak AY-H column (chiral column 2) (15 cm × 3 cm i.d., 5 μm; 15 cm × 3 cm i.d., 5 μm,); Chiralpak AD-H column (chiral column 3); (25 cm × 0.46 cm i.d., 5 μm, 15 cm × 0.46 cm i.d.)	SFC, (40% MeOH, 3 mL/min); HPLC different MP (ethanol/diethylamine (100:0.1 *v*/*v*); 0.2 mL/min) or n-hexane/isopropanol/diethylamine(85:15:0.1 *v*/*v*/*v*); 1 mL/min or n-hexane/ethanol/diethylamine (85:15:0.1 *v*/*v*/*v*); 1 mL/min)	MS	[43]

**Table 2:** Selected applications of HPLC to Food analysis.

Samples	Matrix	Sample Preparation	Technique	Column and Chiral Stationary Phase	Mobile Phase	Detection	References
(*R*,*S*)-triticonazole	Vegetables (tomatos, spinach, cucumber)	Liquid-liquid extraction and Solid phase	HPLC	cellulose tris (3-chloro-4-methylphenylcarbamate) (Lux Cellulose-2); 3-μm particles (250 mm or 4.6 mm i.d.)	-	UV	[44]
Isofenphos-methyl, (*R,S*)-*O*-methyl-*O*-(2-isopropoxcarbonyl)-phenyl-*N*-isopropylphosphora-midothioate	cowpea, cucumber, and pepper	QuEChERS	HPLC-MS/MS	Lux cellulose-3 chiral, column (250 mm × 4.6 mm i.d., 5 μm,	Isocratic, 0.80 mL/min	MS	[46]
Metconazole	Flour	QuEChERS	HPLC	Cellulose 3, 5-dimethylphenylcarbamate Enantiopak OD column	n-hexane-ethanol mixture (97:3, *v*/*v*) at the flow rate of 1.0 mL/min	UV, 220 nm	[47]
Thiols 3-sulfanylhexan-1-ol and its O-acetate, 3-sulfanylhexyl acetate	Wine	Solid phase	HPLC	Lux Amilose 1 and 2, cellulose 1	Gradient, 5 mM aqueous ammonium bicarbonate (A, pH 8.7) and acetonitrile (B)	MS/MS	[48]
8-*O*-4′ type neolignans	Raspberry	-	HPLC	Chiralpak AD-H 250 × 4.6 mm, 5 μm	2-propanol-n-hexane (various ratios)	Polarimeter	[49]
Fungicide pyrisoxazole	Pakchoi, pepper, cabbage	QuEChERS	HPLC	cellulose tris(4-methylbenzoate)-Lux Cellulose-3, 150 mm × 2.0 mm, 3 μm	methanol and water (70:30 *v*/*v*), 0.35 mL/min	MS/MS	[45]
Chloramphenicol	Honey	Liquid-liquid extraction	HPLC	Chiralpak AGP, 3 × 5 mm, 5 μm	Gradient, water with 0.01% acetic acid (A) and methanol with 0.01% acetic acid (B)	MS/MS	[50]
α- and γ-Hexabromocyclododecanes	egg	Soxhlet extraction	HPLC	Permethylated-β-cyclodextrin-Chiral column Nucleosilβ-PM Macherey-Nagel,(GmbH & Co., Düren, Germany), 20 cm × 4 mm × 5 μm	-	MS	[51]
Fluazifop-butyl and fluazifop	tomato, cucumber, pakchoi, rape	Liquid-liquid extraction	HPLC	Cellulose tris(3,5-dichlorophenylcarbamate) Chiralpak IC, 2504.6 mm I.D., 5μm particles	Not reported	MS/MS	[52]
Triacylglycerol	Chicken yolk and meat	Liquid-liquid extraction	HPLC	CHIRALCEL OD-3R, (Daicel Corporation, Tokyo, Japan) 4.6 mm i.d. ×150 mm,	Methanol, 0.5 mL min^−1^	MS	[53]
Amino acids several	Vinegar, milk, kimchi, yogurt	Liquid-liquid extraction	HPLC	CROWNPAK CRIchiral column.	Acetonitrile/ethanol/water/TFA (80/15/5/0.5, *v*/*v*/*v*/*v*)	TOFMS	[54]
Amino acids	Chimchi (fermented vegetables)	Liquid-liquid extraction	HPLC	CROWNPAK CR-I(+) column, 3.0 mm i.d. 150 mm; particle size, 5 μm	Acetonitrile, ethanol, water, and TFA (80:15:5:0.5, *v*/*v*/*v*/*v*)	TOFMS	[55]
Amino acids	Vinegars	Liquid-liquid extraction	HPLC	CROWNPAK CR-I(+) and CR-I(−)(Daicel CPI, Osaka, Japan) (3.0 mm i.d. 150 mm, 5 μm)	acetonitrile, ethanol, water and TFA (80:15:5:0.5, *v*/*v*/*v*/*v*)	MS/MS	[56]
Triazole fungicide (paclobutrazol, myclobutanil, diniconazole, epoxiconazole)	Honey	Solid phase	HPLC	Chiralcel OD-RH column (150 mm × 4.6 mm, 5 μm, daicel, Japan)	ACN/2mM ammonium acetate, 50:42 (*v*/*v*)	MS/MS	[57]
Pesticides	cucumber, tomato, cabbage, grape, mulberry, apple and pear	Magnetic solid phase extraction	HPLC	Chiralpak IG column (250 mm × 4.6 mm, i.d. 5 μm, Daicel, Japan)	ACN/ water containing 5 mmol L^−1^ ammonium acetate and 0.1% (*v*/*v*) formic acid (65:35, *v*/*v*)	MS/MS	[58]
Fungicide prothioconazole and metabolites	Cucumber, pear	QuEChERS and Solid phase	UHPLC and HPLC	Cellulose-tris(4-methylbenzoate) Lux Cellulose-3, 2 or 3, 250 mm × 4.6 mm i.d., 5 μm and 150 mm × 2 mm i.d., 3 μm,	Acetonitrile:water	MS/MS	[59]
(*R*,*S*)-zoxamide	wine	-	UHPLC	Lux Amylose-2 chiral column (150 mm × 2 mm, 3 μm particle	acetonitrile and water (70:30, *v*/*v*), 0.5 mL/min	MS/MS	[60]
Zoxamide	Vegetable (tomato, cucumber), pepper, potato, grape, strawberry	-	UHPLC	Lux Amylose-2, 150 mm × 2 mm, 3 μm particle size	acetonitrile/water (70:30 *v*/*v*), 0.5 mL/min	MS/MS	[61]
Amino acids (derivatized with fluorescein isothiocyanate, FITC) (glutamic acid, aspartic acid, isoleucine, tryptophan, phenylalanine, tyrosine, histidine, proline)	Apple juice	-	Nano-LC (open tubular)	polymerization of 3-chloro-2-hydroxypropylmethacrylate (HPMA-Cl) and ethylene dimethacrylate (EDMA) with bonded β-cyclodextrin; 15 cm and i.d. 75 μm	acetonitrile:methanol:H2O at 0.1% *v*/*v* TFA (85:10:5, *v*/*v*/*v*); flow rate; 800 nL/min	UV, 214 nm	[62]

**Table 3:** Selected applications of GC to Food analysis.

Samples	Matrix	Sample Preparation	Technique	Column and Chiral Stationary Phase	Mobile Phase	Detection	References
TBECH enantiomers 1,2-dibromo-4-(1,2-dibromoethyl)-cyclohexane (α, β, γ, δ) HBCD 1,2,5,6,9,10-hexabromocyclododecane (α, β, γ)	Marine organisms, including 5 mollusk species, 6 crustacean species, and 19 fish species	pressurized fluid extraction	GC	CHIRALDEX B-TA capillary column (30 m × 0.25 mm i.d., 0.12 μm film thickness)	Carrier:40% methane in a helium carrier gas at a constant flow of 1.2 mL/min.	MS	[63]
α-pinene, β-pinene, borneol, camphene, carvone, linalool, limonene, α-terpineol, α-ionene, terpinen-4-ol	Juices (apple, pear, peach, carrot, lemon flesh, orange flesh, orange peel, tangerine flesh), and tangerine peel	Headspace Solid-Phase Microextraction (HS-SPME)	GC	30% 2,3-di-*O*-ethyl-6-*O*-*tert*-butyldimethylsilyl-β-cyclodextrin (diEt-CD) and 30% 2,6-dimethyl-3-*O*-pentyl-β-cyclodextrin (Pentyl-CD) coated	Hydrogen as carrier gas (1.25 mL/min)	MS	[24]
α-pinene, limonene, linalool, β-caryophyllene	Essential oil Thyme	Steam distillation	GC	non-bonded 2,3-di-*O*-methyl-6-tbutyl silyl derivative of β-cyclodextrin AstecChiraldex B-DM column (30 m length 0.25 mm internal, diameter 0.12 μm film thickness)	hydrogen as carrier gas (constant flow of 2.5 mL/min, 8 psistarting column head pressure	Electronic impact ionization and MS	[64]
Pyrethroid insecticide (α-cypermethrin)	tomato, cucumber, rape, cabbage, and pepper	Liquid-liquidextraction	GC	BGB-172 chiral column	N_2_ 100–220 °C	ECD	[65]
Limonene, linalool, α-terpineol and 4-terpineol	Tea	Solid-Phase Microextraction	2D-GC	DB-WAX column (30 m × 0.25 mm i.d., 0.5 μm film thickness) (first) Cyclosil-B column (30 m × 0.25 mm i.d., 0.25 μm film thickness) (second)	helium at 1.2 mL/min	MS	[66]
